# Enhancement of bone regeneration by coadministration of angiogenic and osteogenic factors using messenger RNA

**DOI:** 10.1186/s41232-023-00285-3

**Published:** 2023-06-20

**Authors:** Maorui Zhang, Yuta Fukushima, Kosuke Nozaki, Hideyuki Nakanishi, Jia Deng, Noriyuki Wakabayashi, Keiji Itaka

**Affiliations:** 1 Department of Biofunction Research, Institute of Biomaterials and Bioengineering, Tokyo Medical and Dental University (TMDU), Tokyo, 1010062 Japan; 2 Department of Advanced Prosthodontics, Graduate School of Medical and Dental Sciences, Tokyo Medical and Dental University (TMDU), Tokyo, 1138549 Japan; 3 Department of Oral Implantology, The Affiliated Stomatology Hospital of Southwest Medical University, Luzhou, 646000 People’s Republic of China; 4 Department of Masticatory Function and Health Science, Graduate School of Medical and Dental Sciences, Tokyo Medical and Dental University (TMDU), Tokyo, 113-8549 Japan; 5 Clinical Biotechnology Team, Center for Infectious Disease Education and Research (CiDER), Osaka University, Osaka, 565-0871 Japan

**Keywords:** Bone regeneration, Angiogenesis, mRNA medicine, Vascular endothelial growth factor, Runt-related transcription factor 2, Mandible bone defect, Polyplex nanomicelle

## Abstract

**Background:**

Bone defects remain a challenge today. In addition to osteogenic activation, the crucial role of angiogenesis has also gained attention. In particular, vascular endothelial growth factor (VEGF) is likely to play a significant role in bone regeneration, not only to restore blood supply but also to be directly involved in the osteogenic differentiation of mesenchymal stem cells. In this study, to produce additive angiogenic-osteogenic effects in the process of bone regeneration, VEGF and Runt-related transcription factor 2 (Runx2), an essential transcription factor for osteogenic differentiation, were coadministered with messenger RNAs (mRNAs) to bone defects in the rat mandible.

**Methods:**

The mRNAs encoding *VEGF* or *Runx2* were prepared via in vitro transcription (IVT). Osteogenic differentiation after mRNA transfection was evaluated using primary osteoblast-like cells, followed by an evaluation of the gene expression levels of osteogenic markers. The mRNAs were then administered to a bone defect prepared in the rat mandible using our original cationic polymer-based carrier, the polyplex nanomicelle. The bone regeneration was evaluated by micro-computerized tomography (μCT) imaging, and histologic analyses.

**Results:**

Osteogenic markers such as osteocalcin (*Ocn*) and osteopontin (*Opn*) were significantly upregulated after mRNA transfection. *VEGF* mRNA was revealed to have a distinct osteoblastic function similar to that of *Runx2* mRNA, and the combined use of the two mRNAs resulted in further upregulation of the markers. After in vivo administration into the bone defect, the two mRNAs induced significant enhancement of bone regeneration with increased bone mineralization. Histological analyses using antibodies against the Cluster of Differentiation 31 protein (CD31), alkaline phosphatase (ALP), or OCN revealed that the mRNAs induced the upregulation of osteogenic markers in the defect, together with increased vessel formation, leading to rapid bone formation.

**Conclusions:**

These results demonstrate the feasibility of using mRNA medicines to introduce various therapeutic factors, including transcription factors, into target sites. This study provides valuable information for the development of mRNA therapeutics for tissue engineering.

## Background

Bone defects caused by tumors, trauma, infection, and other diseases remain challenging problems in clinical practice [1, 2]. Despite the capacity for spontaneous healing of bone fractures, a large defect requires prolonged treatment, leading to poor quality of life in patients. In particular, defects in the oral-maxillofacial area cause serious problems in normal facial dynamics and aesthetics [3]. Autologous bone transplantation is regarded as the “gold standard” for maxillofacial bone regeneration, but it is still a significant clinical challenge for surgeons because of limited autologous bone donors, risk of infection, great surgical invasion, and technical difficulty [4]. Additional attempts, such as artificial scaffolds, cell transplantation, and the combined use of growth factors, have been vigorously investigated; however, a standard of care is yet to be established.

Considering the complex processes involved in bone regeneration, angiogenesis, and osteoblast activity are closely interrelated [5]. Angiogenic factors such as VEGF and platelet-derived growth factor are likely to play significant roles in bone regeneration to restore the blood supply by regulating the invasion of sprouting vessels into the ischemic region [6, 7]. In addition, VEGF is directly involved in the osteogenic differentiation of bone marrow mesenchymal stem cells [8]. In embryological experiments, VEGF was also reported to have a spatiotemporal localization profile similar to that of Runx2, an essential transcription factor for osteogenic differentiation and maturation during the osteogenic process [9, 10], suggesting the crucial role of angiogenic-osteogenic coupling in the process of bone regeneration [11].

These studies prompted us to investigate the coadministration of VEGF and Runx2 for the treatment of bone defects. Indeed, we previously demonstrated enhanced bone regeneration by gene therapy using plasmid DNA (pDNA) encoding *Runx2* in a mouse skull bone defect model [12]. Unlike recombinant proteins, pDNAs are available for the simultaneous administration of both transcription and secretory factors at the same time. However, pDNA usually has low transfection efficiency in non-dividing cells. Additionally, the risk of insertional mutagenesis hampers its clinical application.

Now we have another candidate modality, messenger RNA (mRNA), for introducing genes that encode therapeutic proteins. mRNA has the distinct advantage of the negligible risk of integration into the genome [13, 14], making it more suitable for tissue engineering. In addition, we used our original cationic polymer-based carrier, polyplex nanomicelles (see Materials and Methods), to introduce mRNAs. Lipid nanoparticles (LNPs) are the most common carriers of mRNA [15]. LNPs play a crucial role in inducing immune responses [16]. However, in tissue engineering, such inflammatory stimuli should be strictly avoided at the target site. Nanomicelles have already achieved mRNA administration in a less or non-immunogenic manner into the joint [17, 18], intervertebral disc [19, 20], visceral organs [21, 22], and central nervous system [23, 24].

**Fig. 1:**
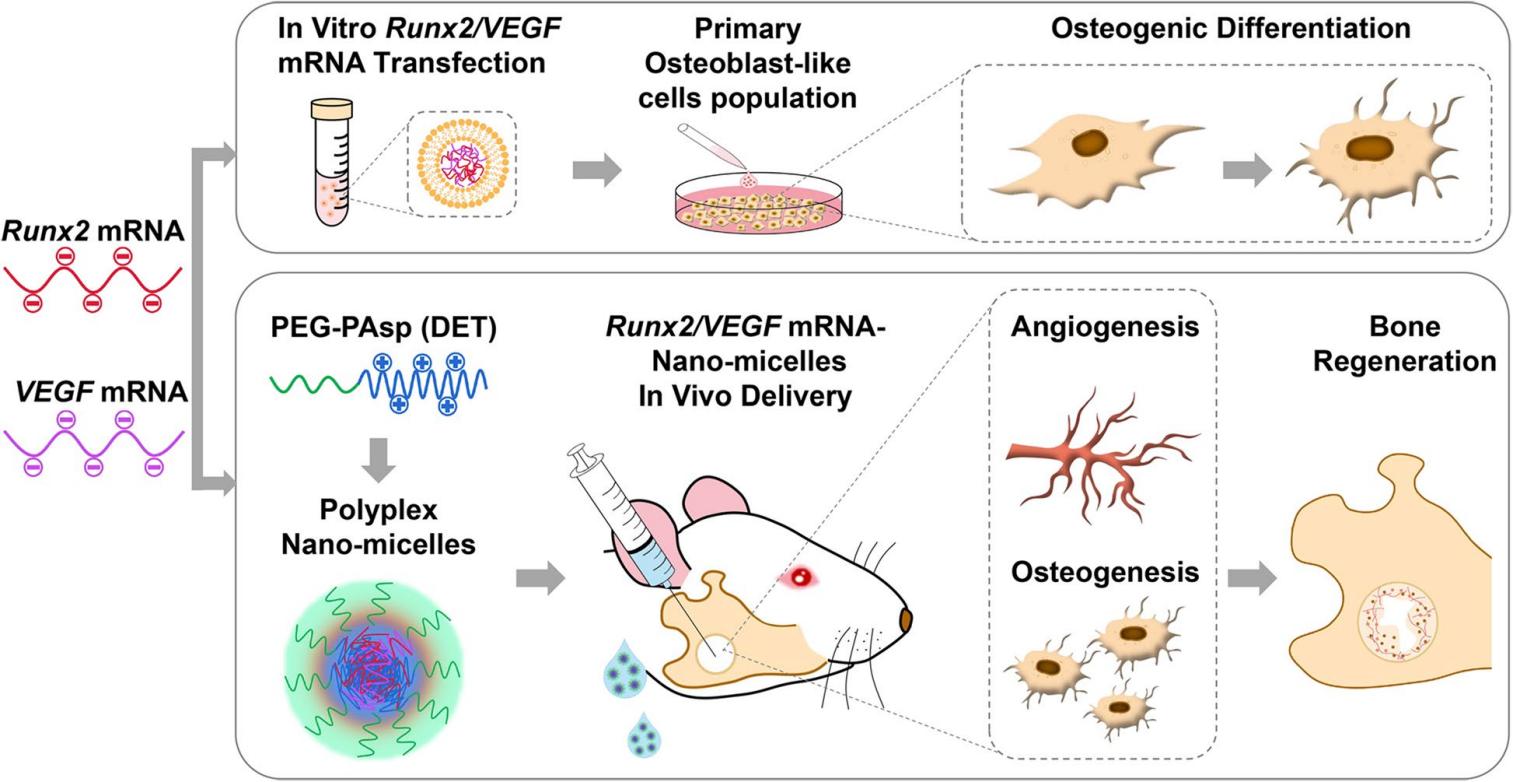
Graphical illustration of mRNA therapeutics for inducing osteogenic differentiation in vitro and bone regeneration in the mandibular bone defect in vivo

In this study, we applied *Runx2* and *VEGF* mRNA to the treatment of a rat model of mandibular defects by administering the mRNAs using polyplex nanomicelles. As shown in the schematic illustration (Fig. 1), the additive effects of *Runx2* and *VEGF* mRNA on osteogenesis were first proven by in vitro transfection into primary osteoblast-like cells. Polyplex nanomicelles loaded with *Runx2* and *VEGF* mRNA were introduced into the mandibular defect area to evaluate bone regeneration, together with detailed analyses of osteogenic markers. This study will lead to new treatments for bone defects using mRNA medicines.

## Materials and methods

### Preparation of mRNAs

Human *Runx2* Vector Builder Japan, Kanagawa, Japan), Human *VEGF* (Thermo Fisher Scientific, Waltham, MA, USA), *Zoanthus sp. green fluorescent protein 1* (*ZsGreen1*) (Takara Bio Inc., Shiga, Japan), *Gaussia luciferase* (*GLuc*) (New England Biolabs Japan, Tokyo, Japan), and *Luciferase*2 (*Luc2*) (Promega Co. Madison, WI, USA) mRNAs were prepared using IVT following our previous studies [13, 19]. The coding region was inserted into the 120-bp poly A/T sequence-containing vector derived from pSP73 vector (Promega, Madison, WI, USA) to control the transcription under the T7 promoter. To prepare linear template DNAs for IVT, each vector was digested by BsmBI-v2 (New England Biolabs Japan). IVT was performed using a MEGAscript T7 Transcription Kit (Thermo Fisher Scientific), CleanCap Reagent AG (TriLink Biotechnologies, San Diego, CA, USA), and N1-Methylpseudouridine-5'-Triphosphate (TriLink Biotechnologies). The prepared mRNA was purified using the RNeasy Mini Kit (Qiagen, Hilden, Germany). The quality and quantity of the IVT mRNA were analyzed using an Agilent 2100 Bioanalyzer chip-based capillary electrophoresis system (Agilent Technologies, Santa Clara, CA, USA) and a Nanodrop One spectrophotometer (Thermo Fisher Scientific, Waltham, MA, USA).

### Primary cell culture

The experimental procedures were approved by the Animal Committee of Tokyo Medical and Dental University. Primary osteoblast-like cells were collected from the cranial bones of neonatal littermates of C57BL/6 J mice (Sankyo Labo Service Co. Tokyo, Japan) and cultured as previously described [12]. In brief, the cranial bone was carefully cut up and digested with 0.1% collagenase X (Wako Pure Chemical Industries Ltd., Osaka, Japan), 0.1% dispase II (Sigma-Aldrich, St. Louis, MO, USA), and 0.05% trypsin–EDTA (Sigma-Aldrich, St. Louis, MO, USA) for 10 min at 37 °C. The suspension was then centrifuged to remove the supernatant, and the precipitate was resuspended and seeded in cell culture flasks (Corning, NY, USA) with alpha-Modified Eagle’s Medium (Sigma-Aldrich, MO, USA) medium containing 10% fetal bovine serum (Sigma-Aldrich, MO, USA) in an incubator with 5% CO_2_ at 37 °C.

### In vitro mRNA transfection

The third-generation primary osteoblast-like cells were seeded in 24-well plates at a density of 4 × 10^4^ cells/well. *Gluc* mRNA (1 μg/well) was transfected using Lipofectamine MessengerMax (Thermo Fisher Scientific, Waltham, MA, USA). Gluc proteins secreted in the culture medium were measured using Renilla-Glo™ Luciferase Assay Reagent (Promega Co. Madison, WI, USA).

### Western blotting

Hela cells (RIKEN BRC, Ibaraki, Japan) were seeded at a density of 2 × 10^5^ cells/well into 6 well-plates. *Runx2* mRNA (2.5 μg/well) or *VEGF* mRNA (2.5 μg/well) was transfected into the cells using Lipofectamine MessengerMax, while the control group was no-transfection treatment. Twenty-four hours later, the total protein was collected from the cells using RIPA Lysis Buffer system (Thermo Fisher Scientific, Waltham, MA, USA). The proteins were separated by sodium dodecyl sulfate–polyacrylamide gel electrophoresis (SDS-PAGE) (BIO-RAD Laboratories, Hercules, CA, USA), and the gels were transferred to polyvinylidene difluoride (PVDF) membranes using the Trans-Blot® Turbo™ Transfer System (BIO-RAD Laboratories, Hercules, CA, USA). Then, the membranes were blocked with 5% skim milk in Tris-buffered saline (TBS) for 1 h at room temperature, followed by incubatation with primary antibodies anti-VEGF (1:1000, ab52917, Abcam, Cambridge, UK) or anti-Runx2 (1:1000, D130-3, MEDICAL & BIOLOGICAL LABORATORIES CO., LTD. Tokyo, Japan) overnight at 4 °C. The membranes were washed by TBS containing 0.05% Tween-20 (TBST) for 10 min and repeated 3 times, and incubated with secondary antibodies (Promega Co. Madison, WI, USA). Repeat washing 3 times by TBST. Finally, the membranes were exposed using iBright™ CL1500 Imaging System (Thermo Fisher Scientific, Waltham, MA, USA) after incubated in SuperSignal™ West Dura Extended Duration Substrate (Thermo Fisher Scientific, Waltham, MA, USA).

### Analyses of osteogenic markers by reverse transcription-polymerase chain reaction (RT-PCR)

For *Runx2* and *VEGF* mRNA transfection, the third-generation primary osteoblast-like cells were seeded in six-well plates at a density of 1 × 10^5^ cells/well. Five groups were set including A: Blank (no mRNA transfection), B: *Luc2* mRNA (2 μg/well), C: *Runx2* (2 μg/well), D: *VEGF* mRNA (2 μg/well), F: *Runx2* (1 μg/well) + *VEGF* (1 μg/well). After the transfection, the total RNA was extracted from the transfected cells on days 3, 7, and 11, followed by an RT-PCR analysis using StepOnePlus Real Time PCR system (Thermo Fisher Scientific, Waltham, MA, USA) to evaluate the expression levels of *Opn, Ocn, β-catenin, lymphoid enhancer binding factor 1* (*Lef1), Osterix* (*Osx*)*, and β-actin* (FastGene™ RNA Basic Kit (Nippon Genetics Co. Tokyo, Japan), ReverTra Ace™ qPCR RT Master Mix with gDNA Remover (TOYOBO Co. Tokyo, Japan) for sample preparation, and AppliedBiosystems™ PowerTrack™ SYBR Green Master Mix (Thermo Fisher Scientific, Waltham, MA, USA) for RT-PCR. The primer sequences are listed in Table 1. The amplification efficiency and quality of the PCR product were examined by melting and standard curves, while the gene cycle threshold (Ct) values from all groups were calculated by the 2^−ΔΔ^Ct method.

**Table 1:** Primer sequences for amplification of genes for RT- PCR

Genes	Symbol	Transcript No	Sequence (5′ → 3′)	Product (bp)
*β-actin*	*β-actin*	NM_007393	F: GTGACGTTGACATCCGTAAAGA R: GCCGGACTCATCGTACTCC	245
*Osteopontin*	*Spp1*	NM_001204201.1	F: CCTGGCTGAATTCTGAGGGAC R: CTTCTGAGATGGGTCAGGCA	185
*Osteocalcin*	*Bglap*	NM_007541.3	F:GAACAGACAAGTCCCACACAGC R:TCAGCAGAGTGAGCAGAAAGAT	79
*β-catenin*	*Ctnnb1*	NM_007614.3	F: TGGTGACAGGGAAGACATCA R: CCACAACAGGCAGTCCATAA	112
*Lef1*	*Lef1*	NM_010703.4	F: GATCCTGGGCAGAAGATGGC R: GCTGTCATTCTGGGACCTGT	195
*Osterix*	*Sp7*	NM_130458.4	F: GATGGCGTCCTCTCTGCTTG R: GCGTATGGCTTCTTTGTGCC	147

### Animal model

All animal experimental procedures were approved by the Animal Committee of Tokyo Medical and Dental University (Approved number: A2021477). All efforts were made to minimize the number of animals and their suffering. Eight-week-old, 260 ~ 280 g weight, male Sprague Dawley rats (Sankyo Labo Service Co., Tokyo, Japan), were housed in a Specific Pathogen Free animal room. Animal surgery to prepare the mandibular bone defect was conducted following previously reported protocols [25]. Briefly, the rats were subjected to general anesthesia with 0.2% isoflurane. Under sterile conditions, a 15 mm incision was made at the mandibular angle. The masseter muscle was sharply separated and the periosteum was bluntly dissected to expose the mandibular angle and lower margin of the mandible. A full-thickness, 4-mm circular defect was created in the mandibular angle using a trephine bur and a low-speed handpiece with 0.9% saline irrigation. The wound was sutured and stratified after achieving hemostasis. The surgical sites showed mild swelling for approximately 24 h after surgery, which gradually subsided within three days.

### Preparation of polyplex nanomicelles

The polyplex nanomicelle is an mRNA carrier formed by the self-assembly of mRNA and a synthetic block-copolymer composed of polyethylene glycol (PEG) and polyamino acid (Poly{N-[N′-(2-aminoethyl)-2-aminoethyl]aspartamide}) (PEG-PAsp(DET)) [26, 27]. The nanomicelles have a diameter of approximately 50 nm, with a core–shell structure surrounded by a dense PEG surface and an mRNA-containing core for the stable retention of mRNA [28, 29]. By preventing nonspecific interactions with other materials, nanomicelles allow smooth tissue penetration in a less or non-immunogenic manner [18].

PEG-PAsp(DET) block copolymers (PEG Mw: 43,000; polymerization degree of PAsp(DET): 63) were synthesized as previously reported [30]. Nanomicelles were formed by mixing solutions of mRNA and block copolymers dissolved separately in 10 mM HEPES buffer. The concentration of mRNA was 300 µg/mL, and that of the block copolymer was adjusted in a way to obtain an N/P ratio (the residual molar ratio of the polycations amino groups to the mRNA phosphate groups) of 3. The solutions of mRNA and block copolymers were mixed at a ratio of 2:1 to form 50 μl solutions.

### In vivo administration of mRNA-loaded polyplex nanomicelles

One week after preparing the mandibular defect, a 50 μl nanomicelle solution containing 10 μg *Luc2* or *ZsGreen1* mRNA was locally injected into the mandibular defect. The luciferase expression was analyzed using In Vivo Imaging System (IVIS) (IVIS®Lumina XRMS III, PerkinElmer Inc. MA, USA). The luminescence radiance was measured after intraperitoneal injection of luciferase substrate with exposure time of 30 s.

For the detection of ZsGreen1 signals, mandibular samples were collected for frozen section and served for histological observation by immunofluorescent staining. The animals were sacrificed under deep isoflurane anesthesia and fixed by transcardiac perfusion with cold 4% paraformaldehyde for 10 min. The mandible was removed, and quickly embedded in Super Cryoembedding Medium (Section Lab Co., Tokyo, Japan) using n-hexane. Four-millimeter horizontal serial slices were sectioned using Kawamoto's film method [31]. The primary anti-ZsGreen1 (Takara Bio Inc., Shiga, Japan) was applied to label the ZsGreen1 protein, followed by Secondary antibodies of Alexa Fluor 488-conjugated goat-anti-rabbit (Invitrogen, Carlsbad, CA, USA). After 1-h incubation, nuclear was stained by 4',6-diamidino-2-phenylindole (DAPI) (Thermo Fisher Scientific, Waltham, MA, USA). The sectional pictures were obtained using a fluorescent microscope (BZ9000; Keyence Co., Itasca, IL, USA) (Fig. 3C, D). From the obtained 4 × picture, three region of interests (ROIs) (20x) were set, two of which (ROI-2 and 3) were set in the defect, and the other (ROI-1) for including the bone edge (Fig. 3E, F, G).

The protein production from *VEGF* mRNA and *Runx2* mRNA was evaluated by immunofluorescent staining, using primary antibodies against anti-Runx2 (1:100) or anti-VEGF (1:100), similarly as described for detecting ZsGreen1 signals.

For analyzing the effect for bone regeneration in vivo, 6 groups were set as follows: Blank (only surgery), HEPES group, *Luc2* mRNA (10 μg), *Runx2* mRNA (10 μg), *VEGF* mRNA (10 μg), *Runx2* (10 μg) + *VEGF* (10 μg) mRNAs (*n* = 3 for each group) (Fig. 5). mRNA administration was conducted weekly for 3 weeks after surgery. At 4 weeks after the surgery, the rats were sacrificed for the following histologic evaluations.

### μCT imaging and structural analysis on the bone defect

The μCT (Micro-CT Lab GX90, Rigaku Co. Tokyo, Japan) was performed to analyze bone formation. Under 0.2% isoflurane anesthesia, rats were placed in μCT to obtain images in the conditions of 90 kV, 160 μA, for 3 min with a Field of View (FOV) of 30 mm. Views are reconstructed in a stack of images containing 512 × 512 × 512 voxels of 59 µm. The image files were constructed using the Digital Imaging and Communications in Medicine format and exported to 3D-BON software (RATOC Systems, CA, USA). Each image was rotated to align the direction of the ROI of a 4 mm-diameter circular bone defect, followed by the calculation of the bone volume (BV), tissue volume (TV), bone mineral content (BMC), and BV/TV ratio in the defect.

### Histologic analysis on osteogenic and angiogenic markers

Similarly as described, the mandible tissues were collected and served for preparing serial sections, followed by Hemotoxylin and Eosin (H&E) staining, Masson–Goldner staining (Sigma-Aldrich, Burlington, VT, USA) (Fig. 7), or immunofluorescent staining (Figs. 8 and 9).

To evaluated osteogenic and angiogenic markers, the sections were immunostained with the primary antibodies: anti-CD31 (1:100, rabbit monoclonal, Abcam, Cambridge, UK), anti-ALP (Santa Cruz Bio. Inc., CA, USA), and anti-OCN (Santa Cruz Bio. Inc., CA, USA). For secondary antibodies, Alexa Fluor 488-conjugated goat-anti-rabbit, or Alexa Fluor 546-conjugated goat-anti-mouse (Invitrogen, Carlsbad, CA, USA) was used. After nuclear staining with 4',6-diamidino-2-phenylindole (DAPI) (Thermo Fisher Scientific, Waltham, MA, USA), sectional images were obtained using an inverted fluorescence microscope (BZ9000). To evaluate the fluorescent signals of CD31, ALP, or OCN, three ROIs were set on each 20 × picture to include bone defect and the newly regenerated bone. The signals were measured three times to three times to be averaged using an imaging software, Image J (National Institutes of Health, USA). The co-stained DAPI signals were used for normalization of the cell number in each ROI, to calculate the percentage of positive area (%). The data in each group was expressed as mean ± S.E.M (Number of rats = 3 per group).

### Statistical analysis

All experimental data were presented as mean ± standard error of the mean and calculated by IBM SPSS19.0 software (IBM Corp., Armonk, NY, USA) with a one-way analysis of variance (ANOVA) and Tukey's post-test. Differences were considered statistically significant at *p* < 0.05.

## Results

### Osteogenic differentiation by *Runx2 *mRNA and *VEGF* mRNA

**Fig. 2:**
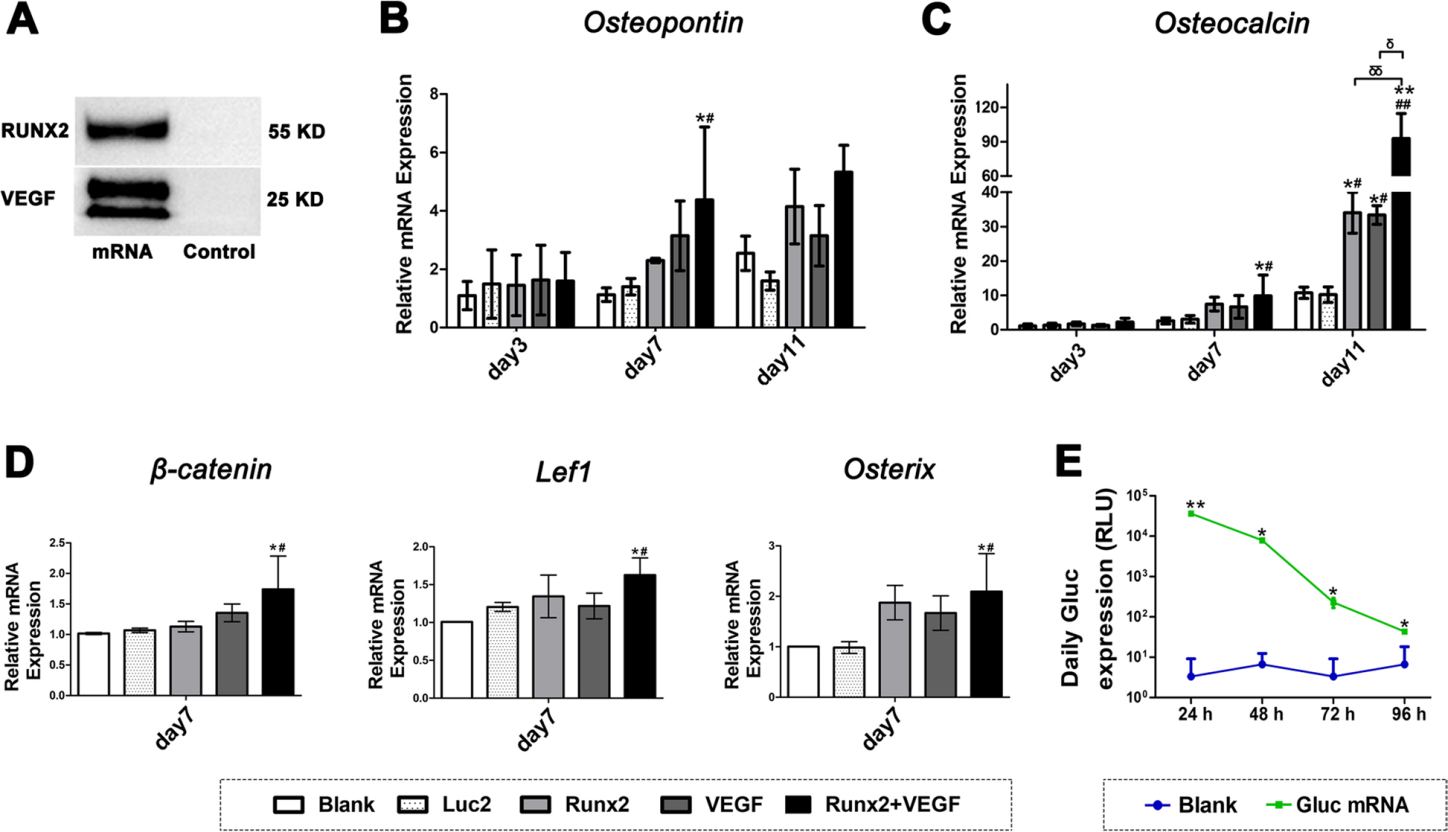
In vitro mRNA transfection toward primary osteoblasts to induce osteogenic differentiation. **A** Western Blotting to confirm Runx2 or VEGF protein production after the mRNA transfection toward HeLa cells. **B**, **C** Relative mRNA expression levels of *Osteopontin* and *Osteocalcin* on days 3, 7, and 11 after the mRNA transfection. The levels were evaluated by RT-PCR. Results were normalized by Blank control on day 3. **D** Relative mRNA expression levels of *β-catenin, Lef1,* and *Osx* on days 7 after the mRNA transfection. Representative data were expressed as mean ± S.E.M. from three independent experiments, and analyzed by one-way ANOVA (post hoc test: Tukey’s multiple comparison test), *n* = 3/group. *:*p* < 0.05, **p < 0.001, compared with Blank group; ^#^*p* < 0.05, ^##^*p* < 0.001, compared with Luc2 group; ^δ^*p* < 0.05, ^δδ^*p* < 0.001, compared with other groups. **E** Time course of Gaussia Luciferase protein production after *Gluc* mRNA (1 μg) transfection toward primary osteoblasts

Firstly, the osteoblastic function of *VEGF* mRNA and *Runx2* mRNA were evaluated by in vitro mRNA transfection. Protein translation from the mRNAs was confirmed by Western Blotting 24 h after transfection into HeLa cells (Fig. 2A). The ability of the mRNAs to induce osteogenic differentiation was evaluated in primary osteoblast-like cells. After the transfection of either *VEGF* mRNA or *Runx2* mRNA or coadministration of the two mRNAs, the expression levels of marker genes for the osteogenic differentiation such as *Opn*, *Ocn, β-catenin*, *Lef1*, and *Osx* were analyzed by quantitative RT-PCR on days 3, 7, and 11. As shown in Fig. 2B-D, the expression levels of the marker genes were generally upregulated by *Runx2* mRNA or *VEGF* mRNA compared to the negative controls. Coadministration of *Runx2* mRNA or *VEGF* mRNA further enhanced their expression levels. Notably, the period of protein production after mRNA transfection is limited to a few days [32]. Indeed, evaluation using an mRNA-encoding secretory Gaussia luciferase, which allowed repeat measurements by collecting samples from the culture medium, showed that protein translation lasted up to four days (Fig. 2E). However, upregulation of the expression levels of marker genes was distinctly detected on day 7, followed by further upregulation on day 11 for *Opn* and *Ocn*. These results suggest that, despite the short period of protein translation from the mRNAs, a single mRNA transfection could trigger the osteogenic differentiation processes, which was detected a week after the transfection. Although the time course of the differentiation remains poorly understood, these in vitro data revealed that *VEGF* mRNA may have a distinct osteoblastic function similar to *Runx2* mRNA, and the combined use of these two factors is expected to further accelerate bone regeneration.

### In vivo administration of mRNA encoding reporter proteins into mandible defect

**Fig. 3:**
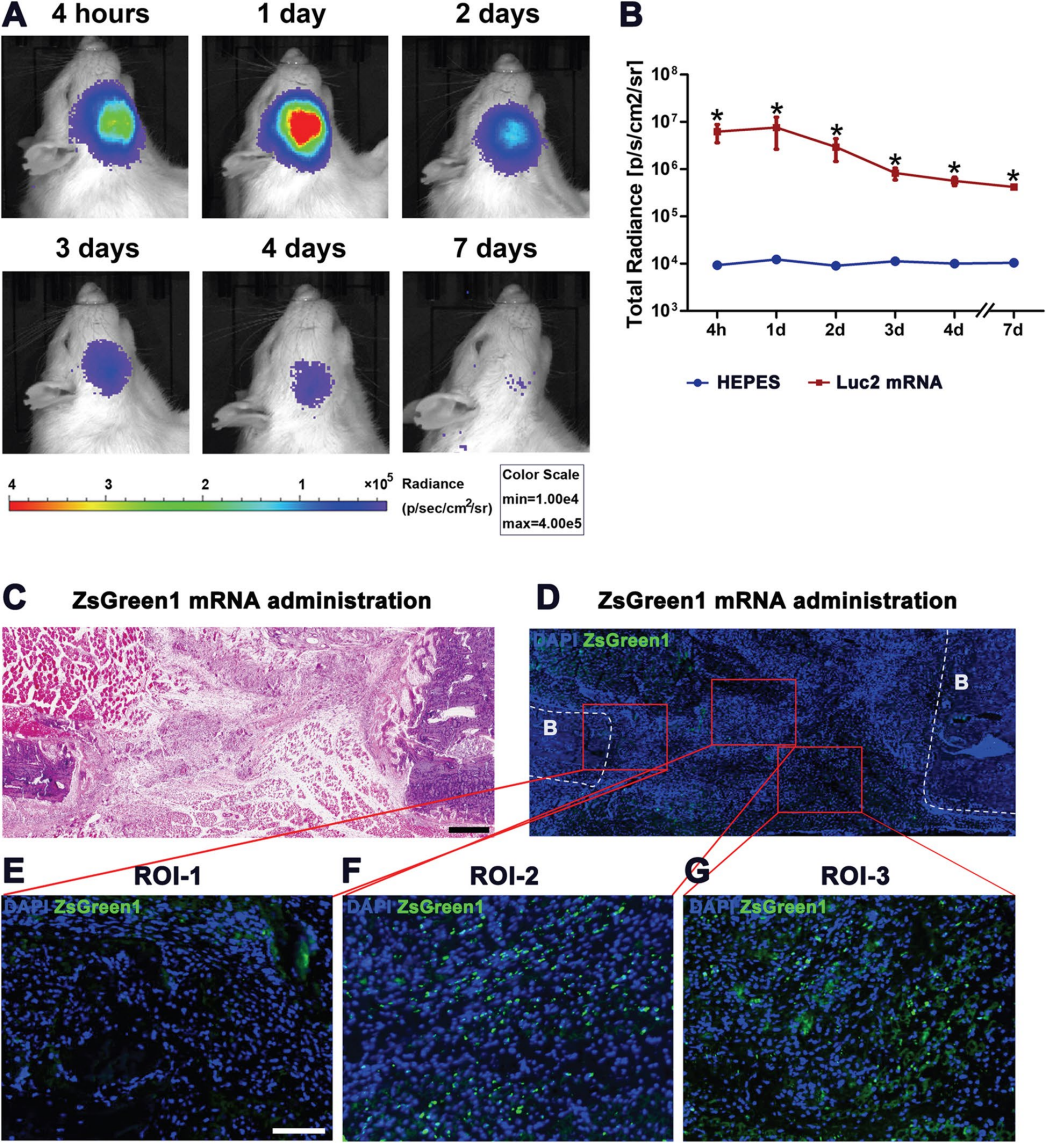
In vivo mRNA administration into the bone defect using polyplex nanomicelles. **A** Luciferase expression visualized by IVIS imaging. 10 µg *Luc2* mRNA was administered into the mandible bone defect. **B** Time course of Luciferase expression. The data of luciferase radiance were expressed as mean ± S.E.M., and analyzed by *t*-test, *:*p* < 0.05. **C**-**G** Immunohistological detection of ZsGreen1 expression 24 h after the administration of *ZsGreen1* mRNA. **C** H&E staining (4 × objective, scale bar = 500 µm), **D** The serial section of (C) with immunohistological staining with anti-ZsGreen1 antibody. B: bone tissue. **E–G** Region of interests (ROIs) to detect ZsGreen1 signals. ROI-1: set for including the bone edge; ROI-2,3: set in the bone defect. (20 × objective, scale bar = 100 µm)

To evaluate the duration and distribution of protein translation in vivo, mRNAs encoding *Luc2* or *ZsGree*n*1* were administered using polyplex nanomicelles into bone defects, which had been created in the rat mandible a week before mRNA administration (see Materials and Methods). Using IVIS imaging, luciferase luminescence was detected as early as 4 h after mRNA administration and peaked at 24 h, followed by a gradual decrease until day 7 (Fig. 3A, B).

The distribution of ZsGreen1 expression was histologically analyzed 24 h after the mRNA administration by immunofluorescent staining. As shown in Fig. 3F and G, the ZsGreen1 signals were well observed in the defect, but almost no signals in the original bone area (Fig. 3E), demonstrating that ZsGreen1 signals were evenly distributed in the defect.

### Bone regeneration in the mandible bone defect by administration of *Runx2/VEGF* mRNAs

**Fig. 4:**
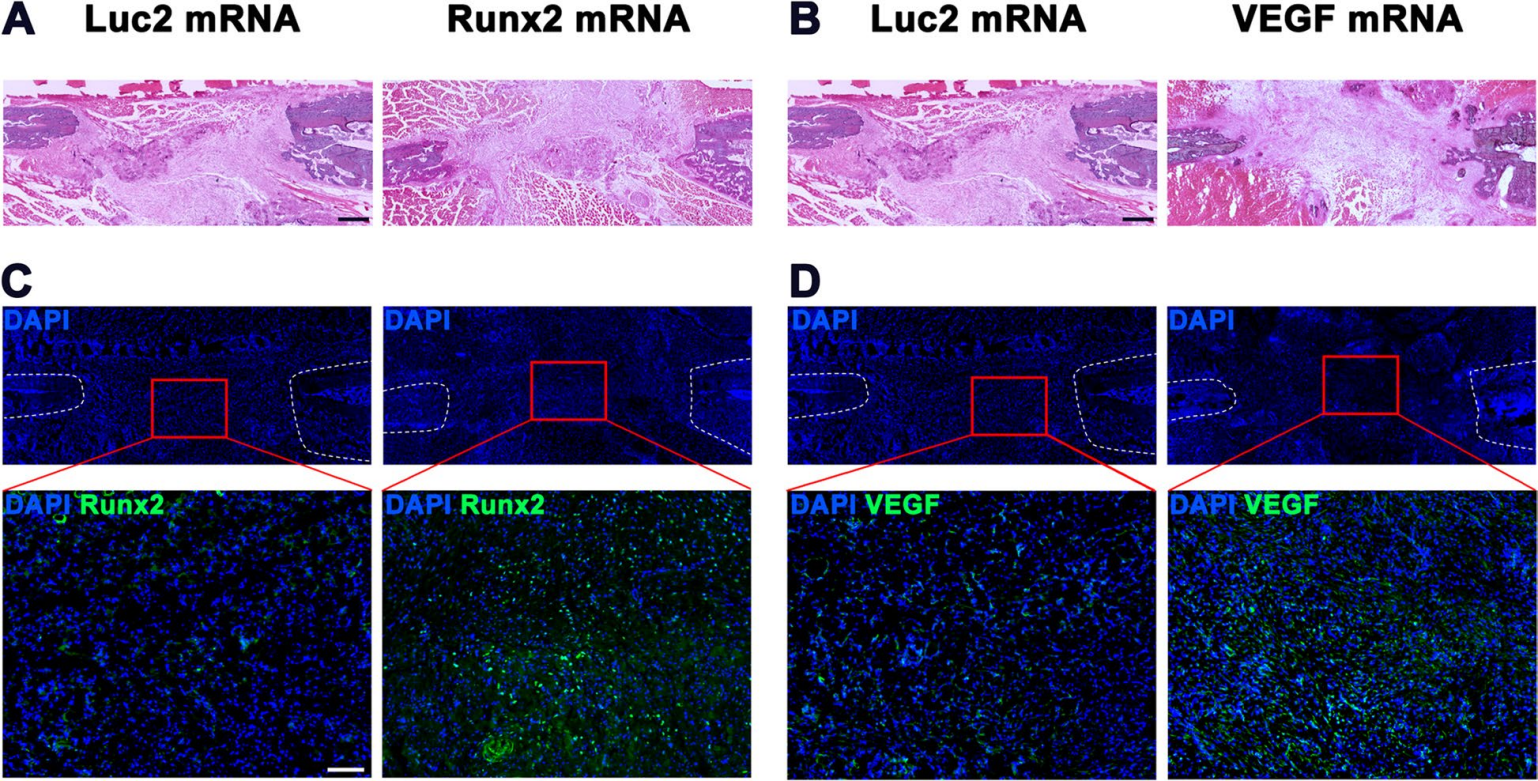
Protein expressions from *Runx2* mRNA or *VEGF* mRNA. **A**, **B** Pictures of the bone defect by H&E staining to define the area of ROIs. (4 × objective, scale bar = 500 µm) (**C**, **D**) Immunofluorescent staining with primary antibodies against anti-Runx2 (C) or anti-VEGF (D). The ROIs were set in the bone defect (20 × objective, scale bar = 100 µm)

**Fig. 5:**
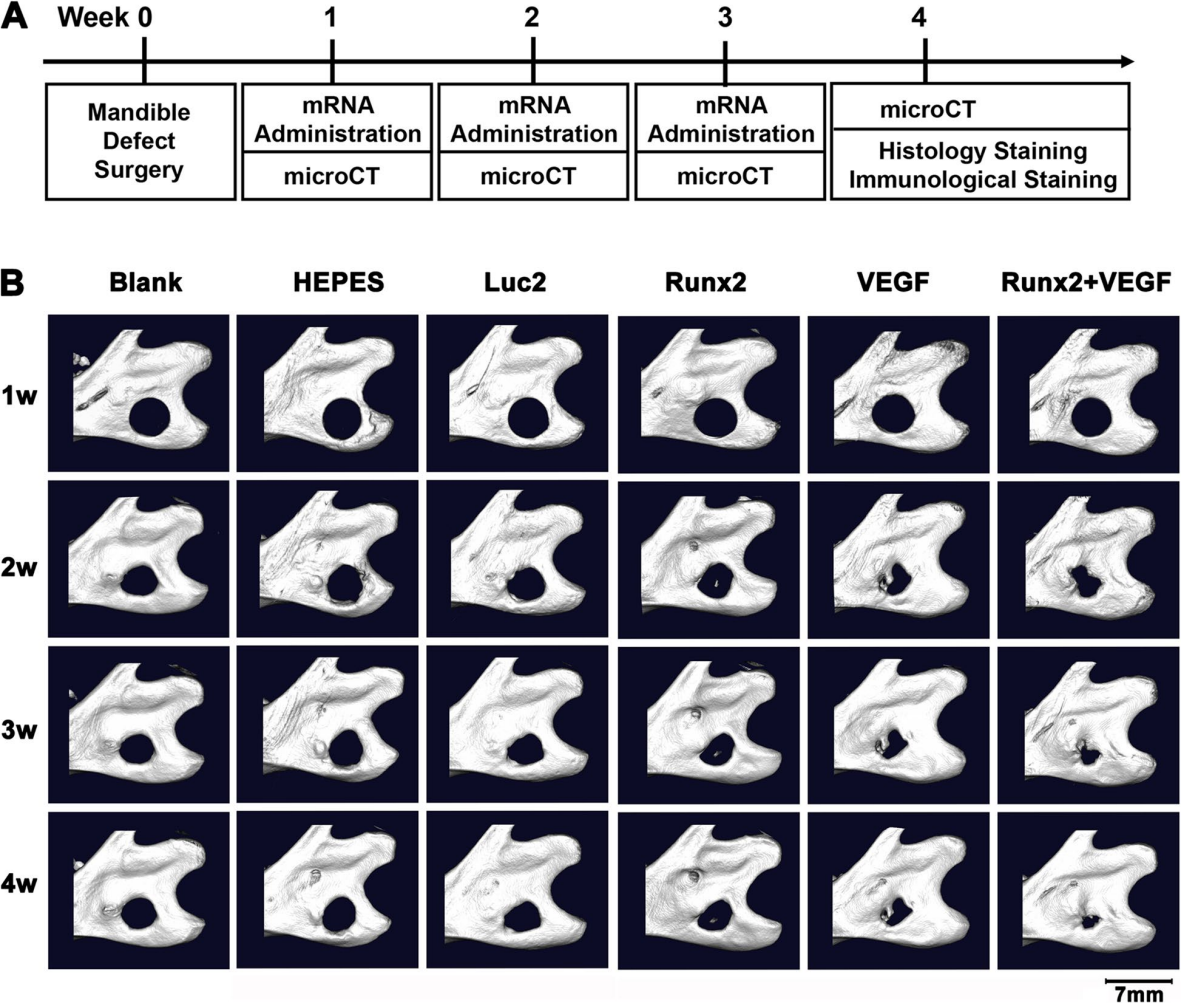
Bone regeneration after the administration of *Runx2* and/or *VEGF* mRNAs into mandible bone defect. **A** Schematic showing the schedule of mRNA administration and μCT imaging. **B** Representative 3D reconstruction images after the mRNA administration: Blank control, administration of only Hepes buffer, administration of *Luc2* mRNA, *Runx2* mRNA, *VEGF* mRNA, and coadministration of *Runx2* and *VEGF* mRNAs. Each mRNA administration was done using polyplex nanomicelle

The efficacy of bone regeneration by Runx2/VEGF mRNAs was evaluated following the protocol shown in Fig. 5A. One week after the surgery, to prepare the mandibular bone defect (see Materials and Methods), weekly mRNA administration was started, with μCT imaging for four weeks after the surgery. The expression of Runx2 or VEGF proteins was confirmed by immunofluorescent staining (Fig. 4), similarly as ZsGreen1 expression.

**Fig. 6:**
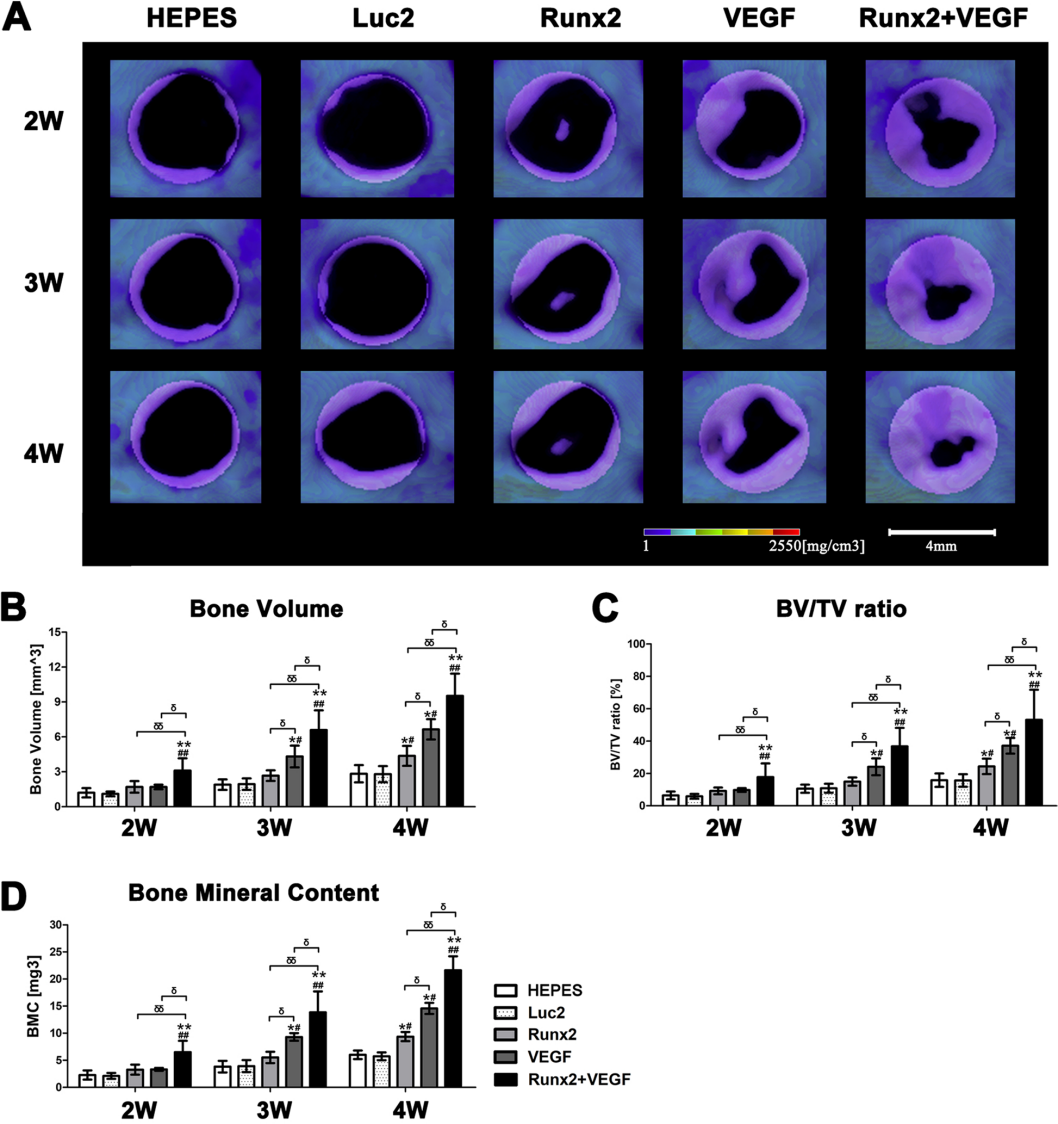
Quantitative analyses of BV, BV/TV ratio, and BMC in the defect. **A** Region of interest (ROI) of a 4 mm-diameter circular bone defect. **B**-**D** Quantification of BV, BV/TV, and BMC. The data were expressed as mean ± S.E.M., and analyzed by one-way ANOVA (post hoc test: Tukey's multiple comparison test), *n* = 3/group. The symbols “*, **, ^#^*,* ^##^, ^δ^, ^δδ^” indicate the significant differences similarly as in Fig. 2

As shown in Fig. 5B, remarkable bone regeneration was observed in the bone defects after the administration of both *Runx2* and *VEGF* mRNAs. Although the defect did not fully recover in four weeks, the combined administration of *Runx2* and *VEGF* mRNAs induced greater bone regeneration than the administration of a single mRNA of *Runx2* or *VEGF.* An imaging study for the quantitative assessment of regenerated bone volume, mineral content, and the ratio of BV to TV also confirmed the tendency of enhanced bone regeneration by the combined use of *Runx2* and *VEGF* mRNAs (Fig. 6).

### Histological analyses of bone-regenerating sites

**Fig. 7:**
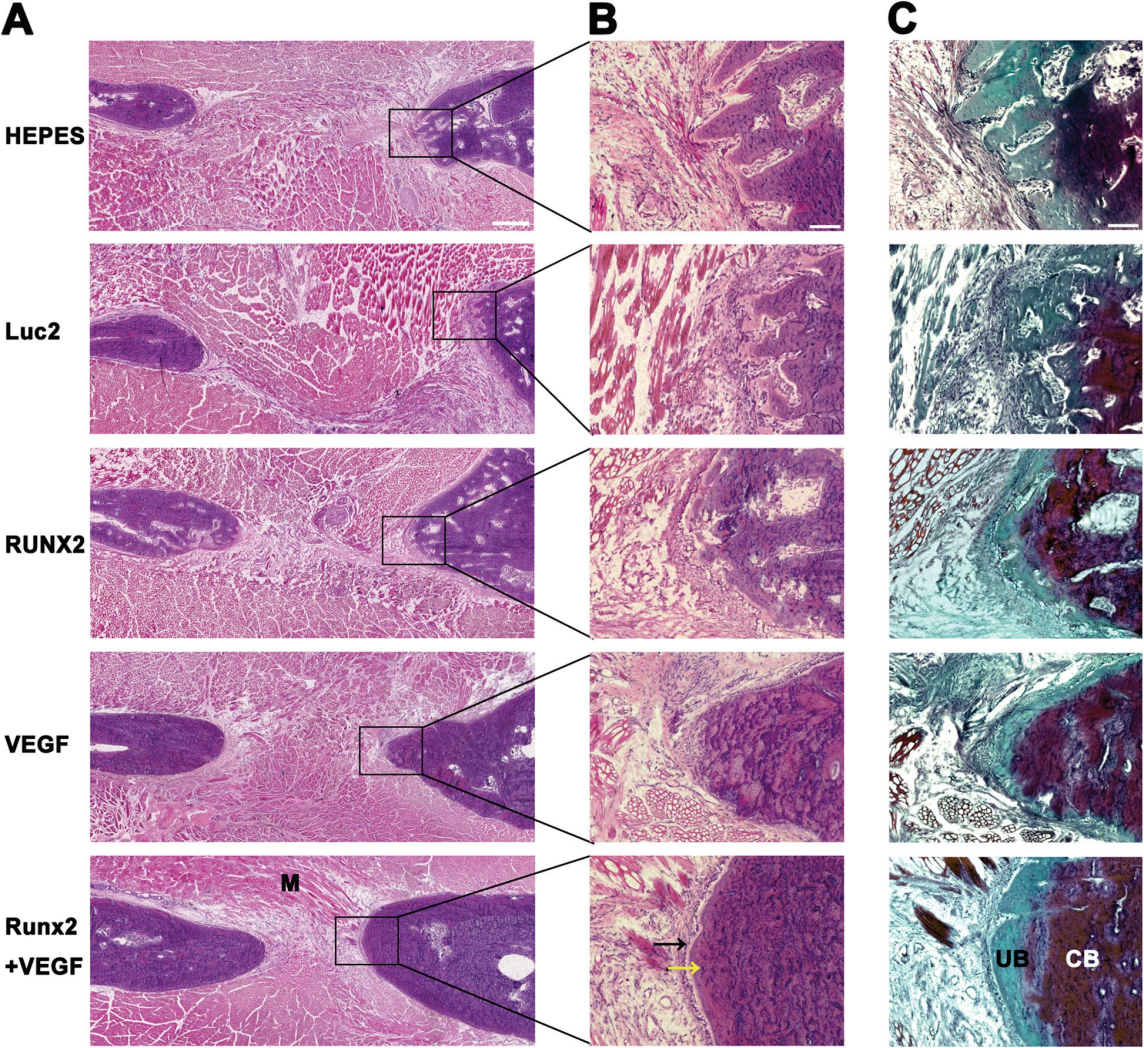
Histologic analysis of the bone defect by H&E staining 4 weeks after mRNA administration. **A** Images in the bone-regenerating sites (4 × objective, scale bar = 500 µm). Although bone defects filled with scar tissue (S) and muscle (M) were still observed in all samples, increased bone mass (B) was observed in the Runx2 and VEGF coadministration group. **B** Images of the bone edge by magnifying the square areas in (A) (20 × objective, scale bar = 100 µm). In the Runx2 and VEGF coadministration group, the bone trabeculae were almost confluent (black arrow), and the bone marrow cavity became smaller (yellow arrow). **C** Masson–Goldner staining on serial sections of (B) (20 × objective, scale bar = 100 µm). In the Runx2 and VEGF coadministration group, newly formed unmineralized bone collagen frames (UB) occupied a smaller area (BD), with more calcified bone areas (CB)

To investigate the mechanism by which mRNAs enhance bone regeneration, histological analyses were performed on bone-regenerating sites four weeks after preparing the bone defect (three weeks after mRNA administration). Although bone defects filled with scar tissue were still observed in all samples, the H&E-stained images showed a large volume of new bone tissue, similar to the original bone observed in the Runx2 and VEGF coadministration group, demonstrating enhancement of the new bone formation process after *Runx2* and *VEGF* mRNA coadministration (Fig. 7A). However, in the HEPES and Luc2 groups, the fiber and muscle tissues covered a large area, while some irregular bone trabeculae were surrounded by bone marrow formed at the margin of the bone defect. The magnified images indicated that in the Runx2 and VEGF coadministration group, the bone trabeculae were almost confluent, and the bone marrow cavity became smaller (Fig. 7B).

Masson–Goldner staining showed that newly formed unmineralized bone collagen frames occupied a smaller area in the Runx2 and VEGF coadministration group (Fig. 7C). The Runx2 and VEGF coadministration group showed more calcified bone areas and properly formed high-density bone tissue. Thus, the results demonstrated that *Runx2/VEGF* mRNA administration not only promoted an increase in bone quantity but also improved bone mineralization (Fig. 7C).

**Fig. 8:**
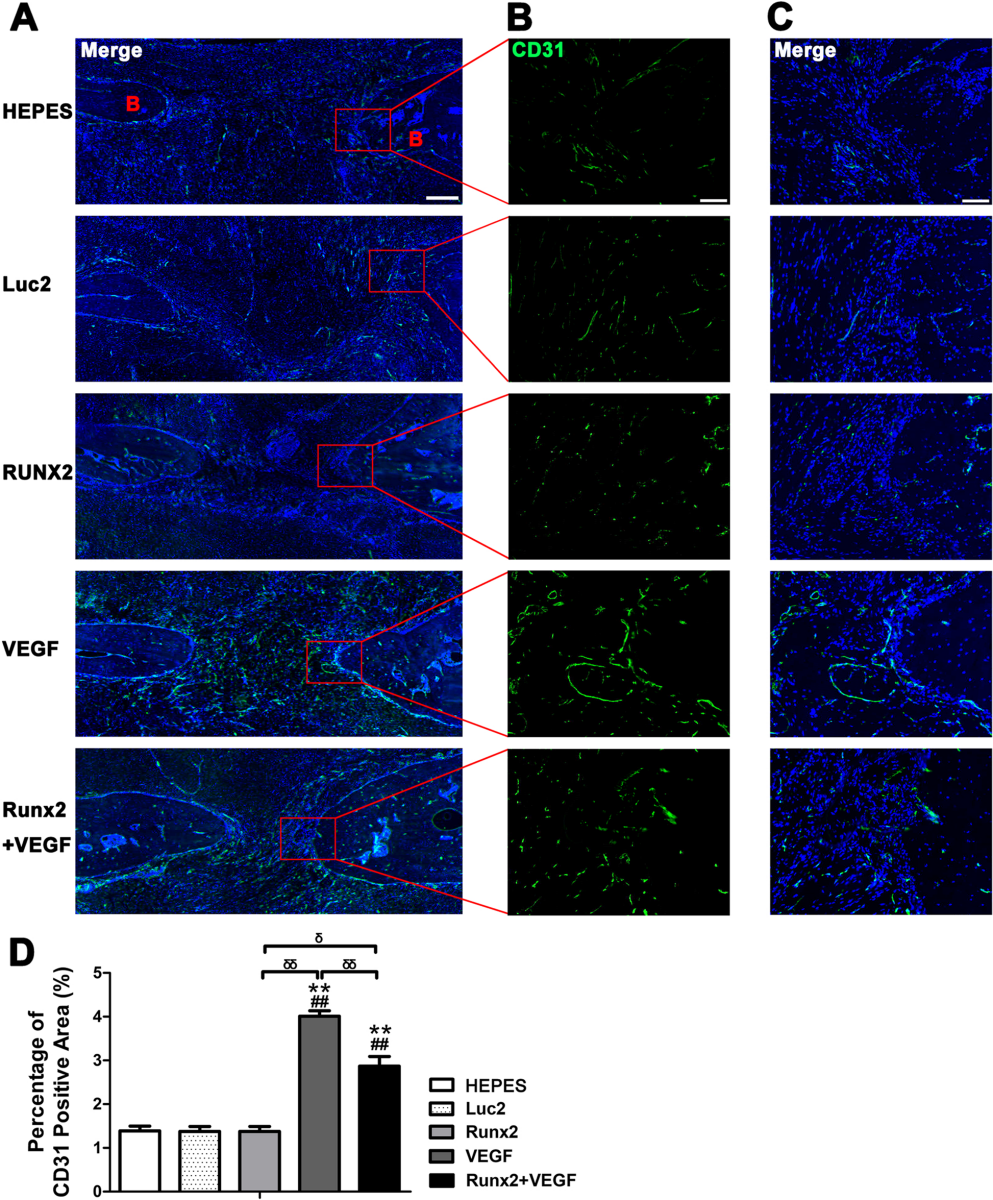
Immunohistological analysis using an antibody against CD31 to detect angiogenesis. **A** Images in the bone-regenerating sites 4 weeks after the surgery to prepare mandible bone defect. Images were merged with those of each serial section after nuclear staining with 4',6-diamidino-2-phenylindole (DAPI) (4 × objective, scale bar = 500 µm). B: bone. **B**, **C** Images of the bone edge by magnifying the square areas in (A) (20 × objective, scale bar = 100 µm). In the groups receiving VEGF mRNA (VEGF and Runx2 + VEGF), CD31-positive signals were widely observed in the defect area. **D** Quantification of CD31 positive area in 20 × objective using Image J software. Data were expressed as mean ± S.E.M., and analyzed by one-way ANOVA (post hoc test: Tukey's multiple comparison test), *n* = 3/group. The symbols “*, **, ^#^*,* ^##^, ^δ^, ^δδ^” indicate the significant differences similarly as in Fig. 2

**Fig. 9:**
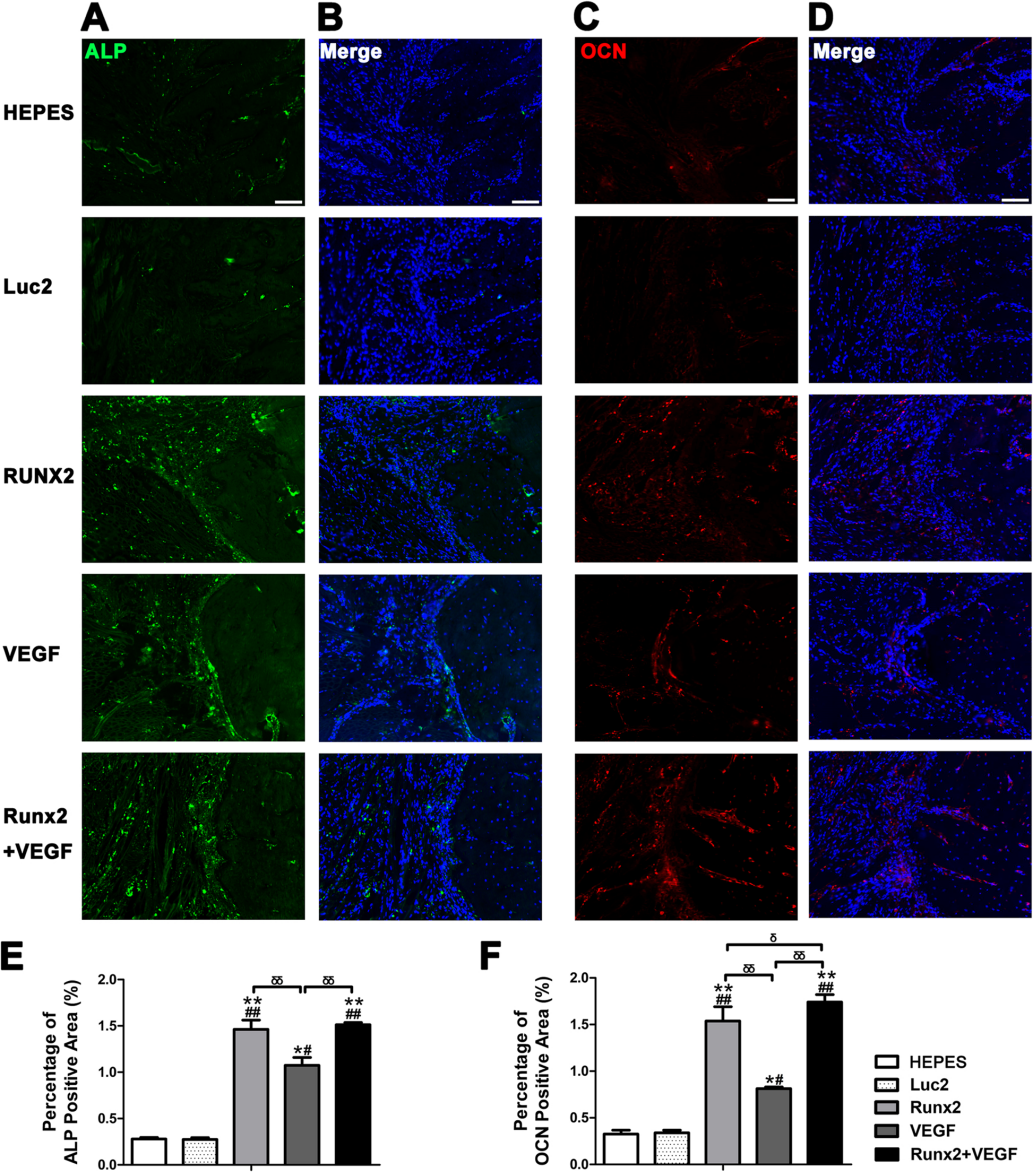
Immunohistological analysis using antibodies against ALP or OCN to evaluate osteoblastic differentiation. **A**, **B** Images stained by ALP antibody, with co-staining by DAPI (B) (20 × objective, scale bar = 100 µm). **C**, **D** Images stained by OCN antibody, with co-staining by DAPI (D). **E**, **F** Quantification of positive areas of ALP (E) or OCN (F), using Image J software. Data were expressed as mean ± S.E.M. and analyzed by one-way ANOVA (post hoc test: Tukey's multiple comparison test), *n* = 3/group. The symbols “*, **, ^#^*,* ^##^, ^δ^, ^δδ^” indicate the significant differences similarly as in Fig. 2

We performed immunohistological analysis using antibodies against CD31 to detect angiogenesis (Fig. 8) and ALP or OCN to evaluate osteoblastic differentiation (Fig. 9). In the groups receiving *VEGF* mRNA (VEGF and Runx2 + VEGF), CD31-positive signals were widely observed in the defect area (Fig. 8), while in other groups, the signals were observed only near the bone border and faintly in the bone marrow. Quantification of the CD31-positive areas further demonstrated the effect of *VEGF* mRNA on the formation of new vessels at the injection site (Fig. 8D).

ALP- and OCN-positive signals were enhanced in the group receiving *Runx2* mRNA, mainly in the bone defect area (Fig. 9A-D). However, it is interesting that the signals were also increased in the group receiving only *VEGF* mRNA compared to the other control groups. Quantification of the positive area of each signal revealed that *Runx2* mRNA induced remarkable upregulation of ALP and OCN, but *VEGF* mRNA may also induce osteoblastic differentiation, even to a lesser extent than *Runx2* mRNA (Fig. 9E, F). Eventually, the combined use of *Runx2* and *VEGF* mRNAs further induced the upregulation of ALP and OCN compared to a single administration of *Runx2* mRNA, which was consistent with the in vivo results of excellent bone regeneration by the combined administration of *Runx2* and *VEGF* mRNAs.

## Discussion

In this study, we demonstrated bone regeneration induced by *Runx2* and *VEGF* mRNA in mandibular bone defects. Compared to the single use of each mRNA, the combined administration of these mRNAs remarkably accelerated bone regeneration. Principally, Runx2 functions as an osteogenic factor [12, 33], and VEGF plays a major role in angiogenesis, which increases the migration of mesenchymal stem cells and precursor cells into the bone defect as well as the supply of nutrients and oxygen required for osteogenesis [34]. However, the role of VEGF may not be limited to angiogenesis but may further affect osteogenic differentiation [8]. Indeed, we found that a single administration of *VEGF* mRNA induced the upregulation of osteogenic markers in vitro (Fig. 2) and considerably enhanced bone regeneration in vivo (Fig. 5), although to a lesser extent than the combined use of *Runx2* and *VEGF* mRNAs. Although it is difficult to determine the relative contributions of these two factors, it was revealed that they act together to enhance bone regeneration in the defect.

In the previous studies of tissue engineering for the bone, the combined use of multiple growth factors including VEGF has been gaining attention, such as BMP-2 and VEGF [35], BMP-6 and VEGF [36], BMP-2, VEGF, and TGF-β1 [37]. Considering the complex processes of bone regeneration, VEGF is a promising candidate factor not only for angiogenesis but also for regulating osteogenic differentiation from migrated cells [38, 39]. In addition, a characteristic feature of this study was the use of the osteogenic transcription factor, Runx2. We have already demonstrated excellent bone regeneration by introducing plasmid DNAs encoding *Runx2* and a constitutively active form of activin receptor-like kinase 6 into an animal model of the bone defect [12]. Unlike secretory proteins such as growth factors, the use of transcription factors can avoid problems such as protein stability and inadequate release profiles (initial burst effect). However, like our previous study, the necessary condition for administering therapeutic transcription factors is that the dosage regimen should be based on a “gene therapy” approach [40, 41].

Therefore, mRNA therapeutics are promising alternatives to gene therapy. Unlike DNA or viral vectors, few studies have investigated mRNA medicines for tissue engineering [18, 42–45], however, the negligible risk of insertional mutagenesis makes mRNAs more favorable for future clinical applications. mRNAs are advantageous in that they can target any cell type, including non-dividing cells. Two or more therapeutic factors can be easily coadministered by mixing different mRNAs.

However, the use of mRNAs for tissue engineering remains difficult. The first issue is the relatively short duration of protein translation from mRNAs. Because tissue regeneration is time-consuming, multiple doses of mRNAs are likely to be necessary. Another limitation of mRNAs is their innate immunogenicity. In particular, when administered with lipid nanoparticles (LNPs) such as mRNA vaccines, the injected site becomes inflamed [15]. Although the mechanism of inflammatory reactions induced by mRNAs and/or LNPs has not been fully investigated [46, 47], it is apparent that these reactions are unsuitable for tissue regeneration and hamper multiple doses of mRNAs.

To overcome these problems, we used polyplex nanomicelles to deliver mRNAs into bone defects. Although the absolute expression levels of the proteins encoded by the administered mRNAs tend to be lower compared with the administration of LNPs (unpublished data), nanomicelles allow repeated mRNA administration safely for various organs, such as the joints and the brain [18, 23]. In this study, we did not fully investigate the mechanism of action of the two factors, e.g. whether they mediated cross-talk with intracellular signalling, or simply worked together in the processes of bone regeneration. The target cell types of the mRNAs and the expression levels required for activating the bone regeneration should also be further investigated. Nevertheless, it should be noted that in our previous studies, we used wild-type mRNAs to obtain therapeutic effects, further highlighting the safety and feasibility of the nanomicelles in a less or non-immunogenic manner.

No artificial scaffolds were used in this study. We designed a rat model of mandibular bone defects such that the space would be surrounded by sufficient soft tissues of the oral and pharyngeal musculature. We then repeatedly injected mRNA into this space. However, for larger bone defects or the bone existing immediately beneath the subcutaneous tissues, such as the skull bone, some scaffolds may be necessary to retain the injected mRNAs in the defect.

## Conclusions

In conclusion, the combined administration of *Runx2* and *VEGF* mRNA using polyplex nanomicelles resulted in excellent bone regeneration in rat mandibular bone defects. These mRNAs induced the upregulation of osteogenic markers in the defect together with increased vessel formation, leading to rapid bone formation. These results demonstrated the feasibility of using mRNA medicines to introduce various therapeutic factors, including transcription factors, into target sites. This study provides relevant information for the development of mRNA therapeutics for tissue engineering.

## Abbreviations

mRNA: Messenger-RNA
IVT: In vitro transcribed
VEGF: Vascular endothelial growth factor
Runx2: Runt-related transcription factor 2
μCT: Micro-computerized tomography
CD31: Cluster of Differentiation 31
ALP: Alkaline phosphatase
OCN: Osteocalcin
OPN: Osteopontin
BMP2: Bone morphogenetic protein 2
PDGF: Platelet-derived growth factor
pDNA: Plasmid DNA
BLA-NCA: β-Benzyl-L-aspartate N-carboxyan hydride
PEG-NH2: α-Methoxy-ω-amino poly ethylene glycol
DET: Diethylenetriamine
HEPES: 2-[4-(2-Hydroxyethyl)-1-piperazinyl]ethanesul fonic acid
PEG-PAsp(DET): Polyethylene glycolpolyamino acid (Poly{N-[N0-(2-aminoethyl)-2-aminoethyl]aspartamide})
ZsGreen1: Zoanthus sp. green fluorescent protein 1
Gluc: Gaussia luciferase
Luc2: Luciferase2
Lef1: Lymphoid enhancer binding factor 1
Osx: Osterix
a-MEM: Alpha-Modified Eagle’s Medium
FBS: Fetal bovine serum
SPF: Specific Pathogen Free
IVIS: In Vivo Imaging System
3D: Three-dimensional
FOV: Field of View
BV: Bone volume
TV: Tissue volume
BMC: Bone Mineral Content
H&E: Hemotoxylin and Eosin
IF: Immunofluorescence
DAPI: 4',6-Diamidino-2-phenylindole
LNPs: Lipid nanoparticles
